# Time for a review of peer review?

**DOI:** 10.1093/jhps/hnx037

**Published:** 2017-09-13

**Authors:** Richard (Ricky) Villar

**Affiliations:** * Editor-in-Chief, Journal of Hip Preservation Surgery

We shot ourselves in the journal foot the other day and, even now, I cannot establish how. The issue was simple. A good colleague, well known to us all, submitted a paper and two reviewers turned it down. It happens. Determined as ever, our colleague submitted the research to another journal who instantly accepted it. We then compounded the injury by citing the now-published work in our subsequent literature review section, What the Papers Say. For some reason, the research appeared better after publication in another journal than when first submitted to *Journal of Hip Preservation Surgery* (*JHPS*) and having read the piece afresh I cannot adequately explain why. Our reviewers had missed a trick, as had I, we had lost a worthy publication and had upset a colleague in the process. Mea culpa.

Or, is it mea culpa? You see, the scenario I cite here is not unusual. *JHPS* is not alone. Indeed, our journal has in my view some of the best peer reviewers in the business. Unrewarded and unsung they have an ability, unsurpassed by many, to take a submission, assess it, frequently improve it, and help the editorial team reach a fair conclusion. At least we feel it is fair, until we do something daft as I have outlined.

Peer review is now under the spotlight and it is not just us. Many would agree that if the peer review process were itself to be peer reviewed the only valid editorial decision would be to revise and resubmit. Birkbeck College in London has recently landed a hefty research grant simply to do just that, to review peer review [1]. It appears that the process is good for weeding out papers that are scientifically weak but not overly fantastic for predicting citations. This is particularly pertinent for *JHPS*. Some of our most highly cited papers received the weakest reviews on submission, whereas some of the least cited received glowing commendations from the start.

As a highly specialist publication we are continually at the edge. We are frequently the first port of call for an author who has something to report but wishes to excite research and comment rather than deliver long-term results. So, because a paper does not have 1-year, 2-year, 3-year results or longer; because an RCT does not have perfect equipoise or, because a control arm simply does not exist, does that render a paper worthless? I would argue not. When you are at the cutting edge, and that is where *JHPS* is positioned, you need to set others thinking as well as reporting results.

The development of hip preservation has been a team approach from the beginning. Yes, there were a few lonely voices at the start, subsequently becoming a veritable army on the advance, but each of us adds what little we can to the work of the majority. That is what has made hip preservation the success it is today. There is no one paper, no one author, no one society, no one editor for that matter, who can claim to rule the roost. Go to any hip preservation meeting and, unless I have misjudged it, you will see practitioners, researchers and colleagues for certain, but overwhelmingly you will see friends united in their aim to move our subspecialty forwards. It is that which has made *JHPS* the success it is today, of that I have no question.

So, I for one am looking forward to the developing debate on peer review, a process that became established in the mid-17th century as a means not so much of identifying good research but of rationing submissions. It was an era when costly hard copy was all that was available. A journal simply could not afford to publish everything it received. Times are now different, thanks to a burgeoning internet and a something-for-nothing culture. Studies have shown that peer review is open to abuse, at times unreliable, and frequently fails to identify fraud [2]. There are now attempts underway to decouple peer review from the publishing process altogether. How can any system of review keep proper pace with the more than one million English-language scientific articles published every year—some would say this is an underestimate—a number that is predicted to double every 9 years?

It is certainly time for the process to be reappraised, especially for those journals that, unlike *JHPS*, do not have a band of reviewers that is worth more than its weight in gold. If you review for *JHPS*, in my view you are the top of the top of the top and words fail me when I try to offer thanks. We are fortunate, many others are not, and yet even we cannot claim to be perfect as my mea-culpa incident shows.

The last issue of *JHPS*, issue 4.2, was truly impressive. The paper by Lund *et al.* [3] describing some of the work being undertaken by the Danish Hip Arthroscopy Register (DHAR) is a lesson to us all. Every one of us should, I believe, be able to accurately report their results, either with a personal register, or a more public one albeit with privacy safeguards attached. Keeping one’s results under wraps is not necessarily the best way of helping our subspecialty advance and the DHAR is thus to be commended. Meanwhile I have often wondered what happens to the sciatic nerve not only during hip arthroscopy, when I cannot see the thing, but at hip replacement, when I can. For this reason, the paper by Hal Martin’s group on sciatic nerve biomechanics during terminal hip flexion is well worth a read. I jest when I say that it has left me terrified of moving the hip at all during surgery, but I certainly know much more about it now than I did before reading the article [4].

And as for this issue of our journal, issue 4.3, I am again spoilt for choice. However, apart from reading the issue from digital cover to cover as you would expect me to recommend, do have a close look at the paper by Degan *et al.* [5] on the risk of failure for primary hip arthroscopy. In our world of more-than-informed consent and lawyers lurking in the shadows, its content is in my view essential. Have a look at the Clinical Vignette, too, that by Atzmon *et al.* [6]. I admit to a total conflict of interest, as only the other day I had to perform a hip arthroscopy in an amputee, not a common situation, and straight to their Vignette I went. Thank you, Atzmon *et al.*, for your tip.

So, as ever, please enjoy this issue of *JHPS*. It is published for you, the hip preservation practitioner, and is filled from cover to cover with pearls. I commend this issue to you in its entirety.

My very best wishes to you all.
